# Assessment of the Second-Order Statically Screened Exchange Correction to the Random Phase Approximation for Correlation Energies

**DOI:** 10.1021/acs.jctc.2c00366

**Published:** 2022-09-23

**Authors:** Arno Förster

**Affiliations:** Theoretical Chemistry, Vrije Universiteit, De Boelelaan 1083, NL-1081 HV, Amsterdam, The Netherlands

## Abstract

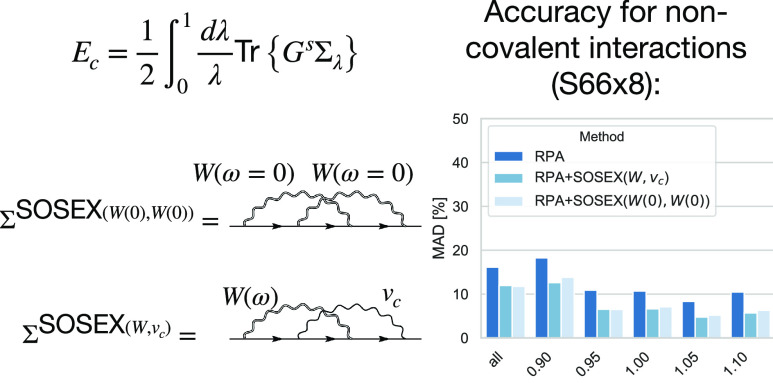

With increasing interelectronic distance, the screening of the electron–electron interaction by the presence of other electrons becomes the dominant source of electron correlation. This effect is described by the random phase approximation (RPA) which is therefore a promising method for the calculation of weak interactions. The success of the RPA relies on the cancellation of errors, which can be traced back to the violation of the crossing symmetry of the 4-point vertex, leading to strongly overestimated total correlation energies. By the addition of second-order screened exchange (SOSEX) to the correlation energy, this issue is substantially reduced. In the adiabatic connection (AC) SOSEX formalism, one of the two electron–electron interaction lines in the second-order exchange term is dynamically screened (SOSEX(*W*, *v*_*c*_)). A related SOSEX expression in which both electron–electron interaction lines are statically screened (SOSEX(*W*(0), *W*(0))) is obtained from the *G*3*W*2 contribution to the electronic self-energy. In contrast to SOSEX(*W*, *v*_*c*_), the evaluation of this correlation energy expression does not require an expensive numerical frequency integration and is therefore advantageous from a computational perspective. We compare the accuracy of the statically screened variant to RPA and RPA+SOSEX(*W*, *v*_*c*_) for a wide range of chemical reactions. While both methods fail for barrier heights, SOSEX(*W*(0), *W*(0)) agrees very well with SOSEX(*W*, *v*_*c*_) for charged excitations and noncovalent interactions where they lead to major improvements over RPA.

## 1 Introduction

The random phase approximation (RPA)^1,2^ has found widespread use in quantum chemistry for the calculation of covalent and noncovalent interaction energies.^3−10^ The direct (particle-hole) RPA can be derived in the framework of the adiabatic connection (AC) fluctuation–dissipation theorem (ACFD)^11−13^ or as a subset of terms in the coupled cluster (CC)^14−18^ singles and doubles (CCD) expansion.^19,20^

Within many-body perturbation theory (MBPT),^21−24^ the RPA is obtained by evaluating the Klein,^25^ or alternatively, the Luttinger-Ward^26^ functional with the self-energy in the *GW* approximation (GWA) using a (noninteracting) Kohn–Sham (KS)^27^ Density functional theory (DFT)^28^ Green’s function.^29,30^ In the GWA,^31^ the self-energy is approximated as the first term of its expansion in terms of a screened electron–electron interaction where screening is usually calculated within a pair bubble approximation^32,24^ Not only for solids but also for larger molecules it becomes decisive to consider screening which is the main reason for the popularity of the *GW* method in solid-state calculations.^24^ The RPA is generally believed to describe long-range electron correlation very accurately Since charge screening is the dominant source of electron correlation in this limit.^12,24^

CC and MBPT based methods describe screening by resummation of certain classes of self-energy diagrams to infinite order.^22,33,34^ The RPA is the simplest first principle method which accounts for these effects and is implemented with  scaling with system size using global density fitting (DF).^35^ Modern RPA (and *GW*) implementations typically use local density-fitting approaches to calculate the noninteracting polarizability,^36−41^ leading to quadratic or cubic scaling in practice, and even effectively linearly scaling implementations (for sufficiently sparse and large systems) have been reported.^42−45^ For these reasons, the RPA is considered a promising method to study weakly correlated large molecules.^4,10,46−48^

At short electron–electron distances, however, charge screening becomes less important for the description of electron correlation and taking into account higher-order contributions to the self-energy via the 4-point vertex function becomes decisive.^49^ The absence of these terms in the RPA leads to Pauli exclusion principle-violating contributions to the electron correlation energy.^50^ As a consequence, total correlation energies are much too high compared to exact reference values.^51,52^

In contrast to RPA, the approximations to the correlation energy of Møller–Plesset (MP) perturbation theory are free of Pauli principle violating terms. Especially MP2 is relatively inexpensive and can be applied routinely to systems with more than 100 atoms even close to the complete basis set limit. However, screening effects are entirely absent in MP perturbation theory and electron correlation is described by HF quasiparticles (QP) interacting via the bare Coulomb interaction instead, neglecting the fact that the interactions between the HF QPs are generally much weaker than the ones between the undressed electrons. This issue is also present in orbital optimized MP2 in which the HF QPs are replaced by MP2 QPs.^53−55^ Therefore, MP2 is a suitable method only for (typically small) systems in which screening effects are negligible. The divergence of MP perturbation theory for the uniform electron gas (see for instance chapter 10 in ref (22) for a thorough discussion) is known at least since early work by Macke^1^ and has been demonstrated later on for metals^56^ and recently also for large, noncovalently bound organic complexes.^48^ The divergence of the MP series for small-gap systems is directly related to this issue since the magnitude of the screening is proportional to the width of the fundamental gap.^57,58^

There have been various approaches to regularize MP2 by an approximate treatment of higher-order screening effects, either using empirical regularizers,^59−69^ diagrammatically motivated modifications^34,70−72^ or attacking the problem from a DFT perspective.^73,74^ Starting from the opposite direction, there have been many attempts to correct the RPA correlation energy expression by adding additional terms to improve the description of short-range correlation. This includes range-separation based approaches,^75−84^ or augmentations by singles contributions.^85−87^ Via MBPT, the RPA can generally be improved upon inclusion of the 4-point vertex in the electronic self-energy, either directly, or indirectly through the kernel of the Bethe-Salpeter equation (BSE) for the generalized susceptibility. Following the latter approach, approximations often start from the ACFD and go beyond the Coulomb kernel in the BSE by adding additional terms, for instance exact exchange (exx) (often denoted as exx-RPA)^88−93^ and higher order contributions,^94−97^ or the statically screened *GW* kernel,^98−100^ but also empirically tuned functions of the eigenvalues of the KS density–density response.^101,102^ Notice that the BSE for the generalized susceptibility reduces to a Dyson equation for the density–density response function which makes local kernels very attractive from a computational perspective.

Instead of relying on the ACFD theorem, beyond-RPA energy expressions can also be introduced directly from approximations to the self-energy beyond the GWA. For instance, in RPAx^103−106^ a local 4-point vertex obtained from the functional derivative of the *local* exact exchange potential calculated within the optimized effective potential method^107−109^ is used in the self-energy. In Freeman’s second-order screened exchange (SOSEX) correction,^110^ the HF vertex (i.e., the functional derivative of the *nonlocal* HF self-energy with respect to the single-particle Green’s function) is included in the self-energy directly but not in the screened interaction.^6,50,86,87,111−113^ Another expression for SOSEX can be obtained by including the static *GW* kernel in the self-energy but not in the density–density response. This possibility has not been explored until recently^114^ and is the main topic of this work.

In our recent work, we have assessed the accuracy of the statically screened *G*3*W*2 correction to the *GW* self-energy for charged excitations.^114^ This correction has first been applied by Grüneis *at al.*(115) to calculate the electronic structure of solids and is obtained by calculating the self-energy to second-order in the screened Coulomb interaction (equivalent to including the full *GW* vertex) and then taking the static limit for both terms. The resulting energy expression fulfills the crossing symmetry of the vertex to first order in the electron–electron interaction. Preliminary results for the correlation energies of atoms have been promising.^114^ This realization of SOSEX is computationally more efficient than AC-SOSEX since no expensive numerical frequency integration is required. Here, we assess the accuracy of this method for bond dissociation, atomization energies, barrier heights, charged excitations, and noncovalent interactions. Our results show that the statically screened SOSEX variant is comparable in accuracy to AC-SOSEX but we observe important differences in the dissociation of diatomic molecules and charged dimers.

The remainder of this work is organized as follows. In section 2 we give a detailed derivation of the different SOSEX energy expressions. After an outline of our computational approach and implementation in section 3, we present and analyze our numerical results in section 4. Finally, section 5 summarizes and concludes this work.

## 2 Theory

The central object of MBPT is the one-particle irreducible (1PI) electronic self-energy Σ. It is the sum of all 1PI skeleton diagrams (diagrams which do not contain any self-energy insertions) of *n*th order in the electron–electron interaction *v*_*c*_. It maps the interacting single-particle Green’s function *G* to its noninteracting counterpart *G*^(0)^ by means of Dyson’s equation,^116^
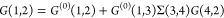
1Space, spin, and imaginary time indices are collected as 1 = (***r***_1_, σ_1_, *iτ*_1_). One can always switch between imaginary time and imaginary frequency using the Laplace transforms^117^
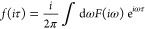
2and
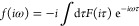
3In (1), *G* = *G*_1_ is defined by
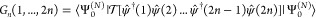
4Here,  is the ground state of an *N*-electron system,  is the time-ordering operator, and  is the field operator. Σ is given by
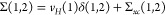
5where the second term on the right hand side (r.h.s.) can be written as
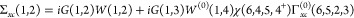
6For a detailed deviation we refer to the Supporting Information. We note that Maggio and Kresse^118^ and Martin et al.^24^ used a similar expression. Equation 6 combines several quantities: These are the particle–hole irreducible 4-point vertex (i.e., the sum of all diagrams contributing to the full 4-point vertex which cannot be cut into parts by removing a particle and a hole line),^119^
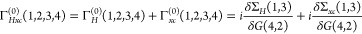
7the noninteracting generalized susceptibility,
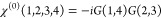
8and the screened (bare) Coulomb interaction *W* (*W*^(0)^). These quantities are related by the Dyson equation
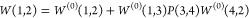
9with
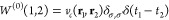
10given in terms of the bare coulomb interaction *v*_*c*_ and the reducible polarizability
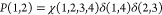
11with
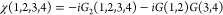
12χ is related to its noninteracting counterpart χ^(0)^ by a Bethe-Salpeter equation (BSE),^119,120^
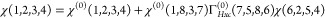
13which reduces to a Dyson equation for the polarizability *P* when the xc-contribution to the 4-point vertex is set to zero. One can then also introduce the irreducible polarizability *P*^(0)^ as
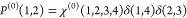
14Using this quantity, (9) can also be written as
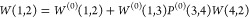
15Note that the equations above are completely equivalent to Hedin’s equations.^31^ Their form given here has the advantages that the BSE appears explicitly and that only 2-point or 4-point quantities occur. Therefore, the resulting equations are invariant under unitary transformations of the basis, as has for instance been pointed out by Starke and Kresse.^121^ or in ref (122).

The xc-contribution to the self-energy defined in (6) can also be obtained as the functional derivative
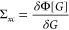
16Φ is a universal functional of the interacting *G* and is defined by^24,26,123^
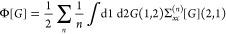
17As for instance discussed in refs (30 and 123), if this expression is evaluated with a noninteracting Green’s function one directly obtains the exchange-correlation energy from it. A suitable noninteracting Green’s function *G*^*s*^ can be obtained from *G*^(0)^ by
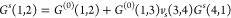
18where
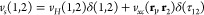
19and with *v*_*xc*_ being a KS xc-potential mixed with a fraction of HF exchange and τ_12_ = τ_1_ – τ_2_. The correlation energy
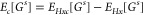
20is then given by^30^
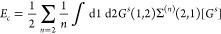
21The *Hx* contribution to the electron–electron interaction energy is obtained as
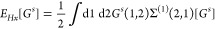
22In the case where *G*^*s*^ is the Hartree–Fock (HF) Green’s function, (22) is the HF expression for the Hartree and exchange energy.

In the GWA, the self-energy (6) is approximated as Σ ≈ Σ_*H*_ + *iGW*. *W* is typically calculated within the RPA which consists in approximating  in the BSE (13). Making both approximations and using eqs 9 and 14, the RPA exchange-correlation energy
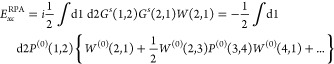
23is obtained.^123^ Isolating the exchange contribution to the Hartree-exchange energy,
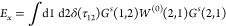
24we obtain the RPA correlation energy
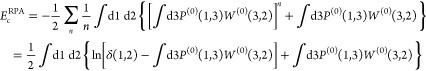
25and using (2) as well as the symmetry of the polarizability on the imaginary frequency axis, its well-known representation due to Langreth and Perdew^12^ is obtained,
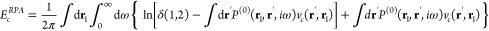
26In this work, we are interested in approximations to the self-energy beyond the GWA. It follows from the antisymmetry of Fermionic Fock space that *G*_2_ needs to change sign when the two creation or annihilation operators in (4) are interchanged. This property is known as the crossing symmetry.^124^ In the RPA, the crossing symmetry is violated which leads to the well-known overestimation of absolute correlation energies. However, when the 4-point vertex is approximated by the functional derivative of the Hartree-exchange self-energy the crossing symmetry is fulfilled. We show this explicitly in the Supporting Information.

Approximations to the self-energy in Hedin’s equations always violate the crossing symmetry.^125,126^ However, with each iteration of Hedin’s pentagon, the crossing symmetry is fulfilled up to an increasingly higher order in *v*_*c*_. We can then expect to obtain improvements over the RPA energies expressions by choosing a self-energy which fulfills the crossing symmetry to first order in *v*_*c*_. The easiest approximation to the self-energy of this type is obtained from the HF vertex,
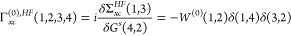
27Using this expression in (6) with (8) yields the AC-SOSEX contribution to the self-energy.^118,127^ We first notice that within the pair bubble approximation for *W*, (6) becomes
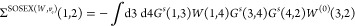
28where we have indicated the screening of the electron–electron interaction in the SOSEX expression in the superscript on the l.h.s. of (28). Here we have used the identity *Wχ*^(0)^ = *W*^(0)^χ in (6) (see Supporting Information) which is only valid if *W* is calculated within the RPA. Using the *GW* self-energy in (7), to first order in *W*^(0)^ (ignoring the variation of *W* with respect to *G*) the screened exchange kernel is obtained,
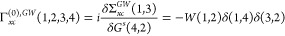
29The resulting self-energy is the complete second-order term in the expansion of the self-energy in terms of the screened electron–electron interaction,^31^
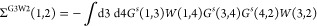
30and contains the AC-SOSEX self-energy.

**Figure 1:**
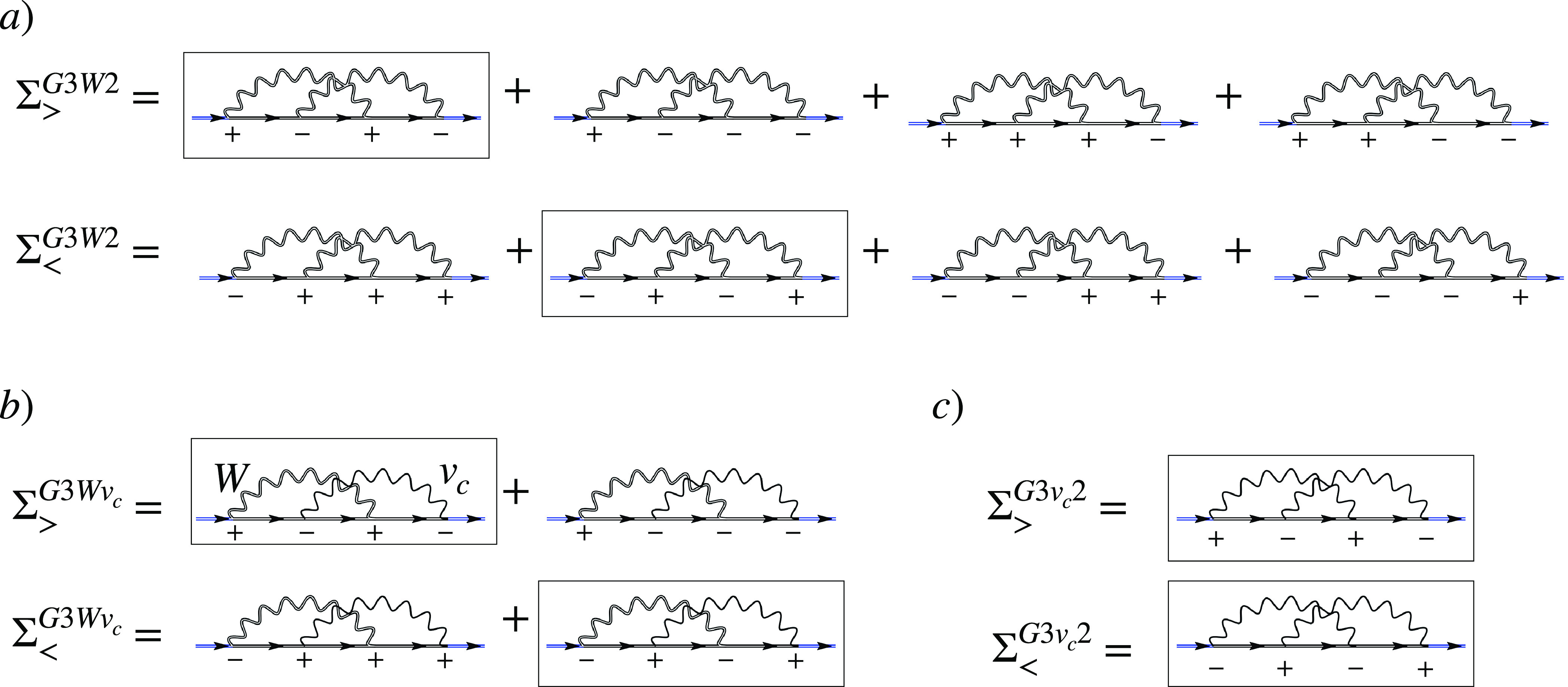
Diagrammatic representation of the different contributions to the second order exchange (SOX) term. Pluses and minuses denote the different branches on the Keldysh contour. The double and single wiggly lines are screened and bare electron–electron interactions, respectively: (a) greater and lesser contributions to the full *G*3*W*2 self-energy term; (b) greater and lesser components of the SOSEX self-energy; (c) greater and lesser components of the MP2 self-energy. The *G*3*W*2 self-energy in the static approximation is equivalent to c), with the bare electron–electron interaction lines replaced by the statically screened ones. The black parts of the diagrams are the contributions to the self-energy only which, combined with the blue lines, yield the corresponding single-particle propagator.

The *G*3*W*2 self-energy can be decomposed into eight skeleton diagrams on the Keldysh contour,^128^ but the AC-SOSEX self-energy only into four.^129^ Diagrammatically, this is shown in Figure 1 panels a and b, respectively. In practice, the evaluation of the resulting energy expression requires a double frequency integration to be performed, while the evaluation of the AC-SOSEX energy only requires a single frequency integration. Since the computation of the AC-SOSEX term is already quite cumbersome, the complete *G*3*W*2 energy expression is therefore not a good candidate for an efficient beyond-RPA correction. Instead, we take the static limit in both *W* in (30) to arrive at a self-energy expression similar to AC-SOSEX,
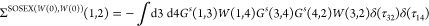
31whose diagrammatic form is shown in Figure 1c). Due to the presence of the two δ-functions, only two out of the eight diagrams of the *G*3*W*2 term remain. This expression is similar to the MP2 self-energy, with the only difference that the bare electron–electron interaction is replaced by the statically screened one. However, the resulting expression for the correlation energy will be different due to the factors  in (17). Using (9), eq 31 can be written as
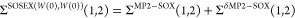
32with the first term being the second-order exchange (SOX) term in MP2 and with the remainder accounting for the screening of the electron–electron interaction. Defining
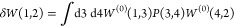
33it can be written as
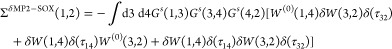
34In the same way one can see that the statically screened *GW* vertex contains the HF vertex. The same is obviously true for all other flavors of SOSEX, and therefore all of them fulfill the crossing symmetry of the full 4-point vertex to first order in the electron–electron interaction. Therefore, all of these approximations compensate the overestimation of the electron correlation energy in the RPA.

In contrast to the RPA which is efficiently evaluated in a localized basis, beyond-RPA energies are most easily formulated in the molecular spin–orbital basis  in which the time-ordered KS Green’s function is diagonal,
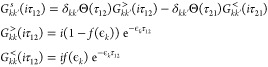
35The ϵ_*k*_ denote KS eigenvalues which are understood to be measured relative to the chemical potential μ, and *f*(ϵ_*k*_) denotes the occupation number of the *k*th orbital. One can now obtain energy expressions analogous to (26). For example, inserting the AC-SOSEX self-energy (28) into (21), we obtain
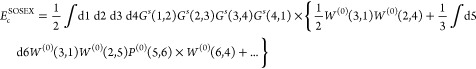
36In contrast to the RPA energy expression, the terms in this equation cannot be summed exactly due to the presence of the 1/*n*-terms. However, defining
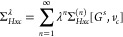
37we can rewrite (21) as an integral over a coupling constant λ,
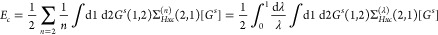
38Therefore, (37) becomes
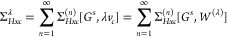
39where *W*^(λ)^ is defined as in (15), with *W*^(0)^ replaced by *λW*^(0)^. Defining
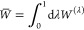
40and
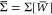
41the correlation energy becomes
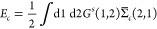
42The integral in (40) needs to be computed numerically, but converges typically very fast when Gauss-Legendre grids are employed.^87^ In ref (130) a trapezoidal rule for the solution of this integral has been used and also ref (3) suggests that this choice is often suitable for the calculation of correlation energies within the RPA and beyond. This choice is very well justified for weakly correlated systems for which the adiabatic connection is approximately a straight line.^131,132^ Below, we will assess the effect of such approximate coupling constant integration on absolute and relative correlation energies for noncovalent interactions. Notice that using a trapezoidal rule (42) reduces to
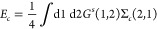
43and when the statically screened *G*3*W*2 self-energy (31) is used in this expression, the energy expression of ref (114) is obtained. When additionally both *W*(0) are replaced by *v*_*c*_, (43) gives the MP2 correlation energy (evaluated with *G*^*s*^).^30^

Using (42), simple expressions for the AC-SOSEX energy in the basis of KS orbitals are obtained. With eqs 28, 35, and 42 we have
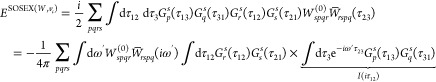
44In going from the second equations, we have used (2) to transform *W* to the imaginary frequency axis. The integral over τ_3_ can be evaluated by splitting it at τ_1_ and using the definition of the KS Green’s function (35),
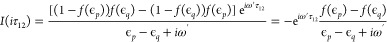
45The remaining integral over τ_12_ is
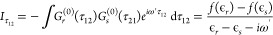
46so that the correlation energy becomes
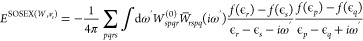
47Each of the numerators can only give a nonvanishing contribution if one of the two occupation numbers are zero. If the difference of the occupation numbers is −1, we simply flip the sign in the denominator. Without loss of generality we can then decide that the indices *r* and *p* belong to occupied and the indices *s* and *q* to virtual single-particle states. Equation 47 then becomes
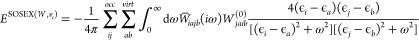
48For a closed-shell system we can also sum over spins which gives us an additional factor of 2. The resulting expression is then equivalent to the one of ref (87). In the spin–orbital basis, the SOSEX(*W*(0), *W*(0)) correlation energy is obtained from (30) and (35) as
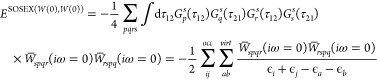
49This is the expression we have introduced in ref (114). It is completely equivalent to the exchange term in MP2 with the bare electron–electron interaction replaced by the statically screened, coupling constant averaged one. Both RPA+SOSEX variants can be understood as renormalized MP2 expressions and allow for a clear diagrammatic interpretation. In the next section, we briefly outline our implementation of these expressions, before we proceed by assessing their accuracy for correlation energies in section 4.

## 3 Technical and Computational Details

All expressions presented herein have been implemented in a locally modified development version of the Amsterdam density functional (ADF) engine of the Amsterdam modeling suite 2022 (AMS2022).^133^ The noninteracting polarizability needed to evaluate (26) and (15) is calculated in imaginary time with quadratic scaling with system size in the atomic orbital basis. The implementation is described in detail in ref (39). In all calculations, we expand the KS Green’s functions in correlation consistent bases of Slater-type orbitals of triple- and quadruple-ζ quality (TZ3P and QZ6P, respectively).^134^ All 4-point correlation functions (screened and unscreened Coulomb interactions as well as polarizabilities) are expressed in auxiliary basis sets of Slater type functions which are usually 5 to 10 times larger than the primary bases. In all calculations, we use auxiliary basis sets of *VeryGood* quality. The transformation between primary and auxiliary basis (for the polarizability) is implemented with quadratic scaling with system size using the pair-atomic density fitting (PADF) method for products of atomic orbitals.^135,136^ For an outline of the implementation of this method in ADF, we refer to ref (137). eq 26 is then evaluated in the basis of auxiliary fit functions with cubic scaling with system size. Equations 48 and 49 are evaluated with quintic scaling with system size in the canonical basis of KS states. This implementation is completely equivalent to the canonical MP2 implementation outlined in ref (137).

Equation 40 is evaluated using Gauss-Legendre grids with 8 points, except for the potential energy curves where 20 points have been used. At stretched bonds, the integrands become increasingly nonlinear and a large number of integration points are necessary. As discussed in detail in the Supporting Information, for noncovalent interactions a single integration point does generally suffice and therefore we have used a single integration point only for all calculations for the S66 and S66 × 8 database. In the case of a single λ-point, a trapezoidal rule is used for integration.

Imaginary time and imaginary frequency variables are discretized using nonuniform bases  and  of sizes *N*_τ_ and *N*_ω_, respectively, tailored to each system. More precisely, (2) and (3) are then implemented by splitting them into sine- and cosine transformation parts as
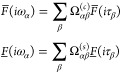
50where  and  denote even and odd parts of *F*, respectively. The transformation from imaginary frequency to imaginary time only requires the (pseudo)inversion of Ω^(*c*)^ and Ω^(*s*)^, respectively. Our procedure to calculate Ω^(*c*)^ and Ω^(*s*)^ as well as  and  follows Kresse and co-workers.^138−140^ The technical specifications of our implementation have been described in the appendix of ref (134).

We use in all calculations grids of 24 points in imaginary time and imaginary frequency which is more than sufficient for convergence.^137^ The final correlation energies are then extrapolated to the complete basis set limit using the relation,^141^
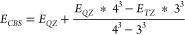
51where *E*_*QZ*_ (*E*_*TZ*_) denotes the total energies at the QZ6P (TZ3P) level. The extrapolation scheme has been shown to be suitable for correlation consistent basis sets but cannot be used for KS or HF contributions.^141,142^ Therefore, we do not extrapolate the DFT energies, but assume them to be converged on the QZ level. Since the basis set error is not completely eliminated with this approach, we also counterpoise correct all energies, taking into account 100% of the counterpoise correction. With these settings, we assume all our calculated values to be converged well enough to be able to draw quantitative conclusions about the performance of the methods we benchmark herein. We use the *VeryGood* numerical quality for integrals over real space and distance cutoffs. Dependency thresholds^39^ have been set to 5*e*^–4^.

All Full configuration interaction calculations have been performed with the code by Knowles and Handy.^143,144^ The 1- and 2-electron integral which are required as input have been generated with ADF.

## 4 Results

### Dissociation Curves

The potential energy curves of small diatomic molecules serve as an important test for electronic structure methods. We first consider molecules with different bonding types for which we were able to calculate FCI reference values: H_2_ is covalently bound, LiH is an ionic molecule, and He_2_ has a very weak, noncovalent bond.

**Figure 2:**
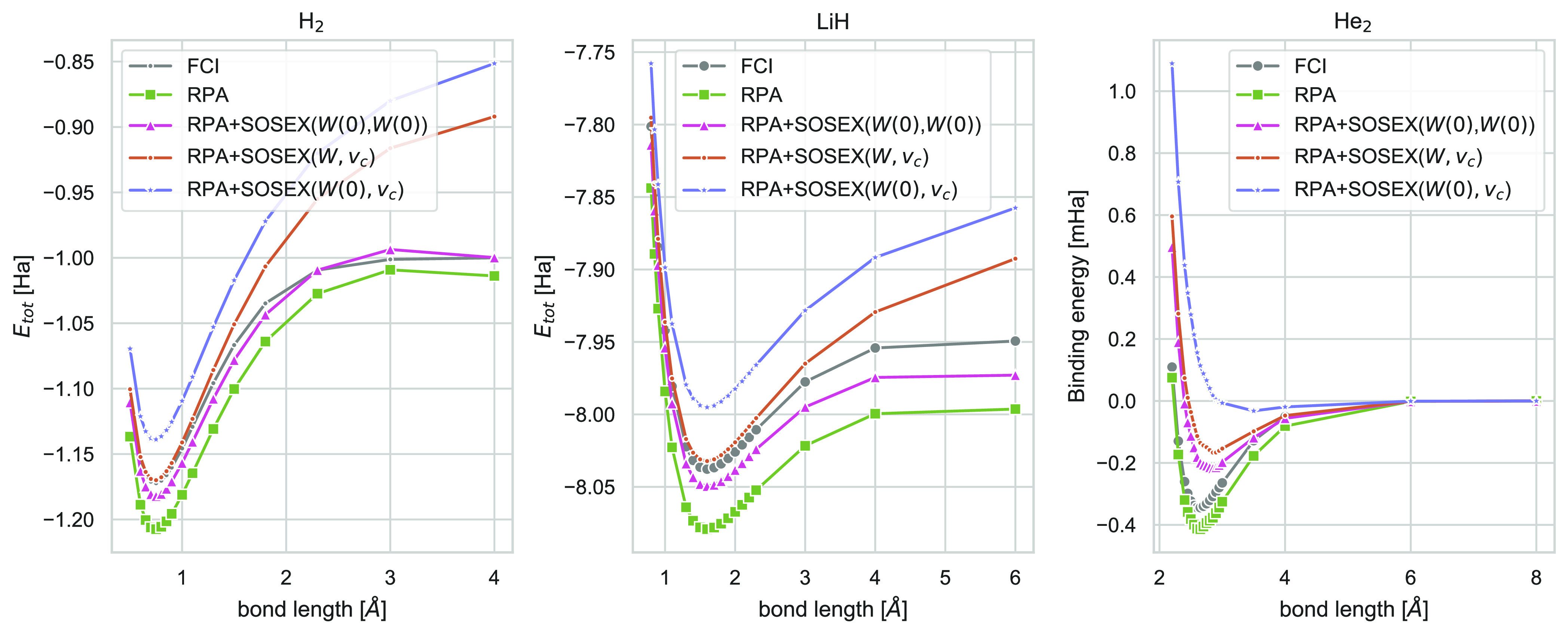
Potential energy curves (in Hartree) of H_2_ (left) and LiH (middle), as well as binding energy (in mHartree) as a function of system size for He_2_ on the right using FCI, RPA@PBE, and different variants of RPA+SOSEX@PBE. For H_2_ and He_2_, all calculations have been performed with the TZ3P basis set. For LiH, all calculations have been performed using the TZP basis set.

The dissociation curve of H_2_ calculated with RPA+SOSEX(*W*(0), *W*(0))@PBE is the red line in Figure 2. Our calculations are not converged with respect to the basis set size but comparison of our dissociation curves calculated with RPA@PBE and RPA+SOSEX(*W*, *v*_*c*_)@PBE to the ones presented in refs (112 and 145) clearly shows that their qualitative behavior is reproduced correctly. It is known that RPA describes the dissociation of covalently bonded molecules qualitatively correctly while RPA+SOSEX(*W*, *v*_*c*_) and other exchange-corrected RPA approaches fail.^91,112,145^ Here we find that also RPA+SOSEX(*W*(0), *W*(0)) dissociates the hydrogen molecule correctly and that the potential energy curve has a similar shape than the RPA one. Henderson and Scuseria have argued that the self-correlation in the RPA mimics static correlation effects^145^ whose good description is necessary to dissociate H_2_ correctly. Therefore, the fact that SOSEX(*W*(0), *W*(0)) to a large extent (see also Table 1 in the SI) but not completely eliminates the RPA self-interaction error explains the similarity to the RPA dissociation curve.

**Table 1:** Equilibrium Bond Length of Selected Molecules^a^

method	H_2_	LiH	He_2_	F_2_	Be_2_
exp.				1.413^146^	2.320^147^
accurate	0.741	1.601	2.626	1.413^146^	2.320^148^
RPA	0.742	1.597	2.632	1.437	2.403
RPA + SOSEX(*W*(0), *W*(0))	0.744	1.605	2.852	1.444	2.424
RPA + SOSEX(*W*, *v*_*c*_)	0.738	1.594	2.871	1.364	
RPA + SOSEX(*W*(0), *v*_*c*_)	0.735	1.599	3.542	1.348	

a All values are in Å. The bond lengths for H_2_, He_2_, and LiH have been calculated using the TZ3P and TZP basis sets to make them comparable to the FCI result. The bond lengths for F_2_ and Be_2_ have been obtained using the QZ6P basis set. All RPA(+SOSEX) calculations have been performed with a PBE Green’s function.

To rationalize this result further, we also calculated the dissociation curve within the static limit of RPA+SOSEX(*W*, *v*_*c*_), RPA+SOSEX(*W*(0), *v*_*c*_) (blue curve). This shows that the screening of the second electron–electron interaction line is responsible for the qualitative differences between SOSEX(*W*, *v*_*c*_) and SOSEX(*W*(0), *W*(0)). It should also be noted that the RPA+SOSEX(*W*(0), *W*(0)) dissociation curve of H_2_ very closely resembles the one calculated by Bates and Furche using the approximate exchange kernel (AXK) correction to the RPA.^94^ SOSEX(*W*(0), *W*(0)) and the AXK kernel have in common that both electron–electron interaction lines are screened. For LiH, we find a similar behavior than for H_2_. For He_2_ (notice that we plotted here the binding energy and not the total energy) we see that all approaches give the correct dissociation limit.

From these potential energy curves, we also extracted the equilibrium bond lengths via cubic spline interpolation. These are shown in Table 1 Around the equilibrium distances, RPA+SOSEX(*W*, *v*_*c*_) generally gives the best energies but this does not necessarily translate into the best bond lengths. For the covalently bound molecules, LiH and F_2_ as well as LiH RPA+SOSEX(*W*, *v*_*c*_) underestimate and RPA+SOSEX(*W*(0), *W*(0)) overestimates the bond lengths. Again, RPA+SOSEX(*W*(0), *W*(0)) behaves qualitatively similar to RPA. For He_2_, both approaches give similar results, while RPA+SOSEX(*W*(0), *v*_*c*_) fails completely. On the other hand, unlike RPA+SOSEX(*W*(0), *W*(0)), RPA+SOSEX(*W*, *v*_*c*_) does predict an unbound Be_2_ dimer.

### Dissociation of Charged Dimers

In Table 2 we investigate the dissociation of four charged dimers by means of the SIE4 × 4 data set.^149^ Here, the self-correlation error of RPA leads to considerable underbinding,^6,112,145^ whereas RPA+SOSEX(*W*, *v*_*c*_) is almost exact,^113^ the remaining error for H_2_ being due to basis set errors as well as the fact that PBE orbitals have been used. Furche and co-workers have observed a catastrophic failure of RPA+SOX for  and (150), and also SOSEX(*W*(0), *W*(0)) considerably deteriorates the RPA results for those systems. Only for , one finds that the partial cancellation of the RPA self-correlation leads to small improvements over RPA.

**Table 2:** Errors in kcal/mol for the Charger Dimers in the SIE4 × 4 Benchmark Set Calculated with RPA and Different Variants of RPA+SOSEX^a^

	RPA	SOSEX(*W*, *v*_*c*_)	SOSEX(*W*(0), *v*_*c*_)	SOSEX(*W*(0), *W*(0))
H_2_^+^				
1.0	5.19	0.76	–2.58	3.09
1.25	7.59	–0.26	–5.33	5.19
1.50	11.21	–1.31	–8.23	8.89
1.75	16.15	–2.30	–11.14	14.27
He_2_^+^				
1.0	13.23	0.23	–5.30	14.34
1.25	25.40	–2.84	–12.91	27.56
1.50	40.60	–5.64	–20.32	44.79
1.75	56.76	–7.65	–25.76	63.38
(NH_3_)_2_^+^				
1.0	5.89	15.17	24.91	16.23
1.25	13.00	20.08	36.23	33.50
1.50	20.61	21.89	42.78	50.41
1.75	30.88	15.14	28.73	61.48
(H_2_O)_2_^+^				
1.0	10.19	29.79	51.70	33.79
1.25	20.62	12.16	21.61	38.68
1.50	31.88	2.35	4.58	50.58
1.75	42.08	0.50	5.47	65.61
MAD	21.95	8.63	19.22	33.24

a PBE orbitals have been used in all calculations.

### Thermochemistry and Kinetics

We move on to assess the performance of RPA+SOSEX(*W*(0), *W*(0)) for reaction types which are relevant for thermochemistry and kinetics. Total atomization energies, ionization potentials, and electron affinities as well as barrier heights of different reactions serve hereby as important testing grounds. For this work, we calculated the atomization energies (defined as the total energy of the molecule minus the sum of the energies of the atomic fragments) of the 144 small and medium molecules in the W4-11 data set.^151^ The reference values have been calculated using the highly accurate W4 protocol.^152^ For barrier heights, we use the BH76 database which is a compilation of the HTBH38^153^ and NHTBH38^154^ databases for barrier heights by Truhlar and co-workers, which are typically used in benchmarks of (beyond-)RPA methods.^5,6,86,87^ The reference values have been calculated with the W2–F12 protocol.^149,155^ To benchmark the performance for ionization potentials and electron affinities we employ the G21IP and G21EA databases by Pople and co-workers and use the original experimental reference values.^156^

To start with, we assess the effect of the Green’s function *G*^*s*^ used to calculate the correlation energies. RPA calculations can in principle be performed self-consistently using a variety of approaches^88,157−164^ (see ref (165) for a review). This is rarely done in practice since self-consistent RPA calculations are computationally demanding and since the resulting energies are often worse than the ones evaluated using a Green’s function from a generalized gradient approximation (GGA) or hybrid calculation.^159^ GGAs like PBE or TPSS are often used to construct *G*^*s*^.^9,48,87^ Using hybrid orbitals can be seen as a pragmatic way to compensate for the lack of self-consistency in the RPA calculation and therefore we assess here whether they lead to improvements over GGA orbitals.

**Figure 3:**
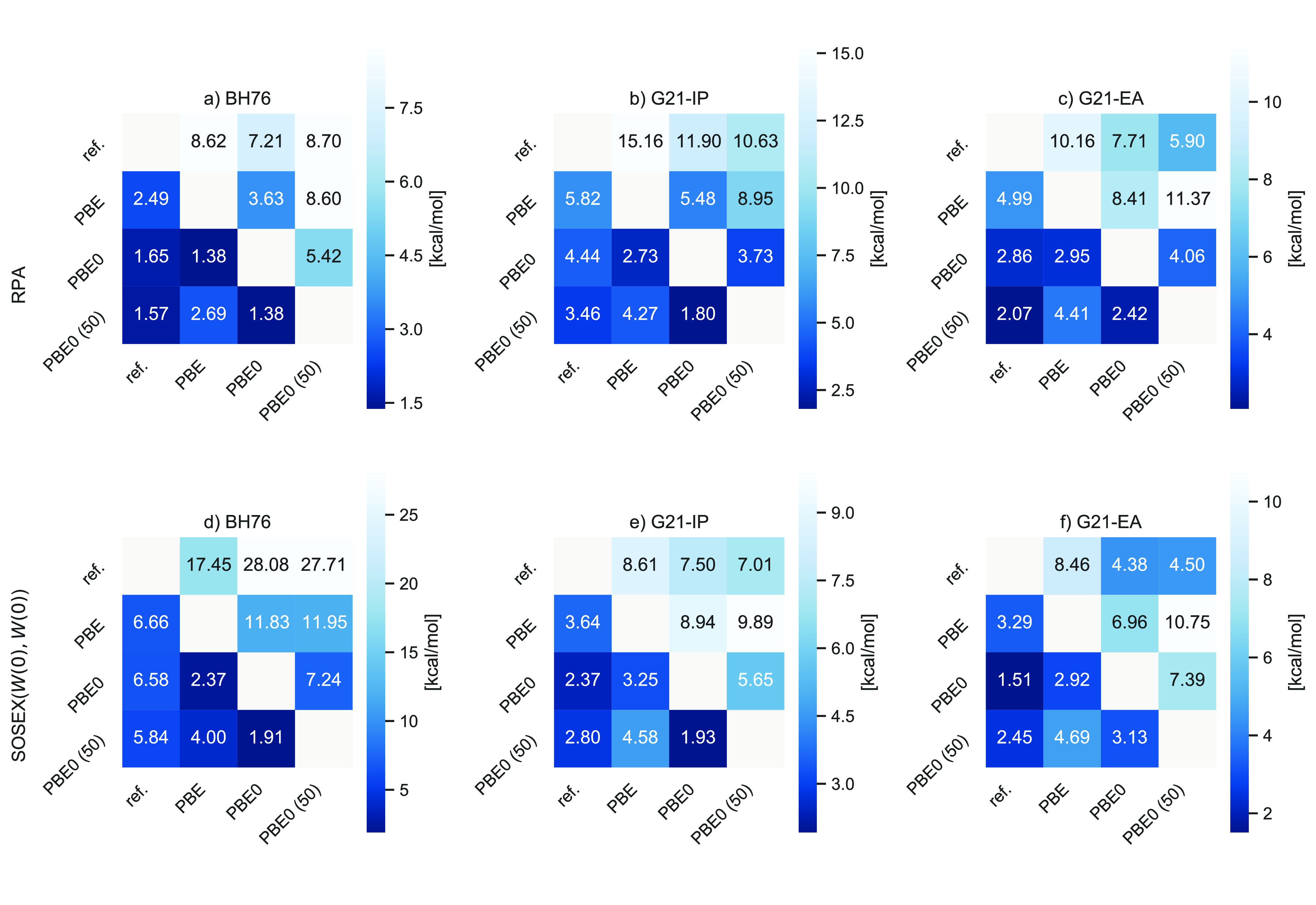
Mean absolute deviations (MAD) (lower triangle in each plot) and maximum deviations (MAX) (upper triangle) with respect to the reference values as well as using different KS Green’s functions as input for BH76 (left), G21-IP (middle), and G21-EA (right) for RPA (top) and RPA+SOSEX(*W*(0), *W*(0)) (bottom). All values are in kcal/mol.

For W4–11, the differences between different starting points are minor, but PBE tends to give the best results. For the BH76, G21IP, and G21EA data sets, we show mean absolute deviations (MAD) and maximum deviations (MAX) with respect to the reference values and with respect to the different starting points in Figure 3. The RPA results generally improve with increasing amount of Fock exchange, while 25% (PBE0) generally seems to work best for RPA+SOSEX(*W*(0), *W*(0)). The differences are often substantial, for instance in case of the RPA barrier heights (Figure 3a) or the RPA+SOSEX(*W*(0), *W*(0)) electron affinities (Figure 3f).

For charged excitations, this observation aligns very well with the experience from *G*_0_*W*_0_ calculations where hybrid functionals with 25–50% are typically a much better starting point than GGAs.^166,167^ However, when *G*3*W*2 corrections are added to the *G*_0_*W*_0_ QP energies, using a hybrid functional with a smaller fraction of exact exchange might often be beneficial.^114,168^ For barrier heights, hybrid functionals with a larger fraction of exact exchange are usually required to obtain qualitatively correct barrier heights,^149,169^ and it therefore is not surprising that hybrid orbitals serve as a suitable starting point for RPA calculations.

**Figure 4:**
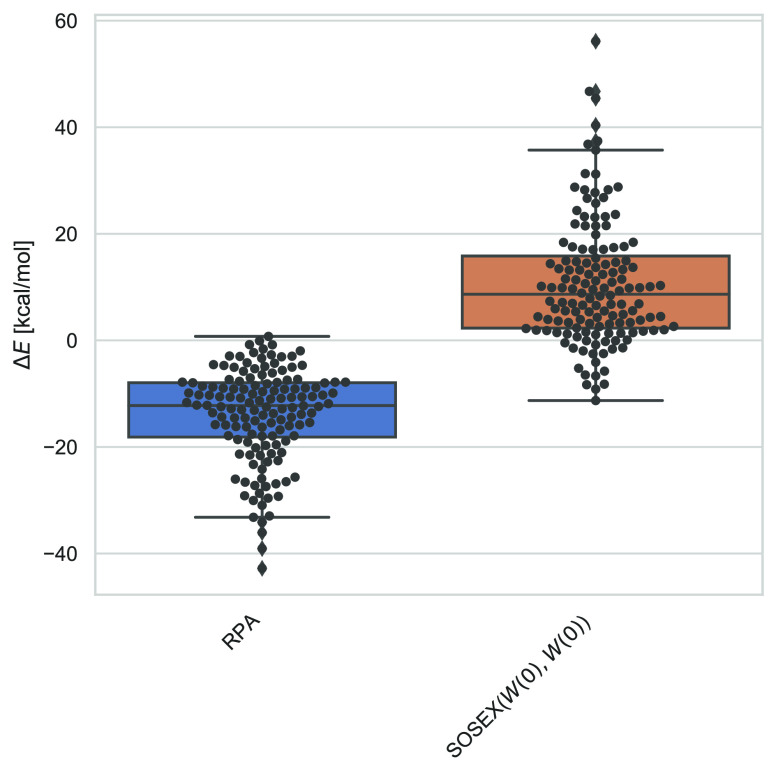
Errors of RPA@PBE and RPA+SOSEX(*W*, *v*_*c*_)@PBE for the atomization energies in the W4–11 data set. Black dots denote the individual data points, and the horizontal line in each box denotes the median deviation. The box contains all data points between the first quartile (*Q*1) and third quartile (*Q*2), and the whiskers are at *Q*1 ± |*Q*1 – *Q*3| (in case of a normal distribution, the whiskers include 99.3% of all data points). All values are in kcal/mol.

Our atomization energies for the W4-11 data set are shown in Figure 4. It has first been observed by Furche^170^ that RPA underestimates atomization energies (indicated here by negative errors). This was confirmed later by Ren at al.^6^ and Paier et al.^86^ for the 55 covalently bound molecules in the G2-I set.^156^ The same holds for RPA+SOSEX(*W*, *v*_*c*_), but compared to RPA the magnitude of the error is reduced on average.^6,86^ We observe here that unlike SOSEX(*W*, *v*_*c*_), the addition of SOSEX(*W*(0), *W*(0)) substantially overcorrects the RPA atomization energies which are now much too high in magnitude.^171^ Adding bare SOX to RPA leads to underestimated correlation energies.^52^ This effect is expected to be more pronounced for the molecule than for the individual atoms since more electrons are correlated in the former. Therefore, RPA+SOX will substantially overestimate atomization energies, and due to underestimated screening of the SOX term in SOSEX(*W*(0), *W*(0)), RPA+SOSEX(*W*(0), *W*(0)) inherits this problem.

**Figure 5:**
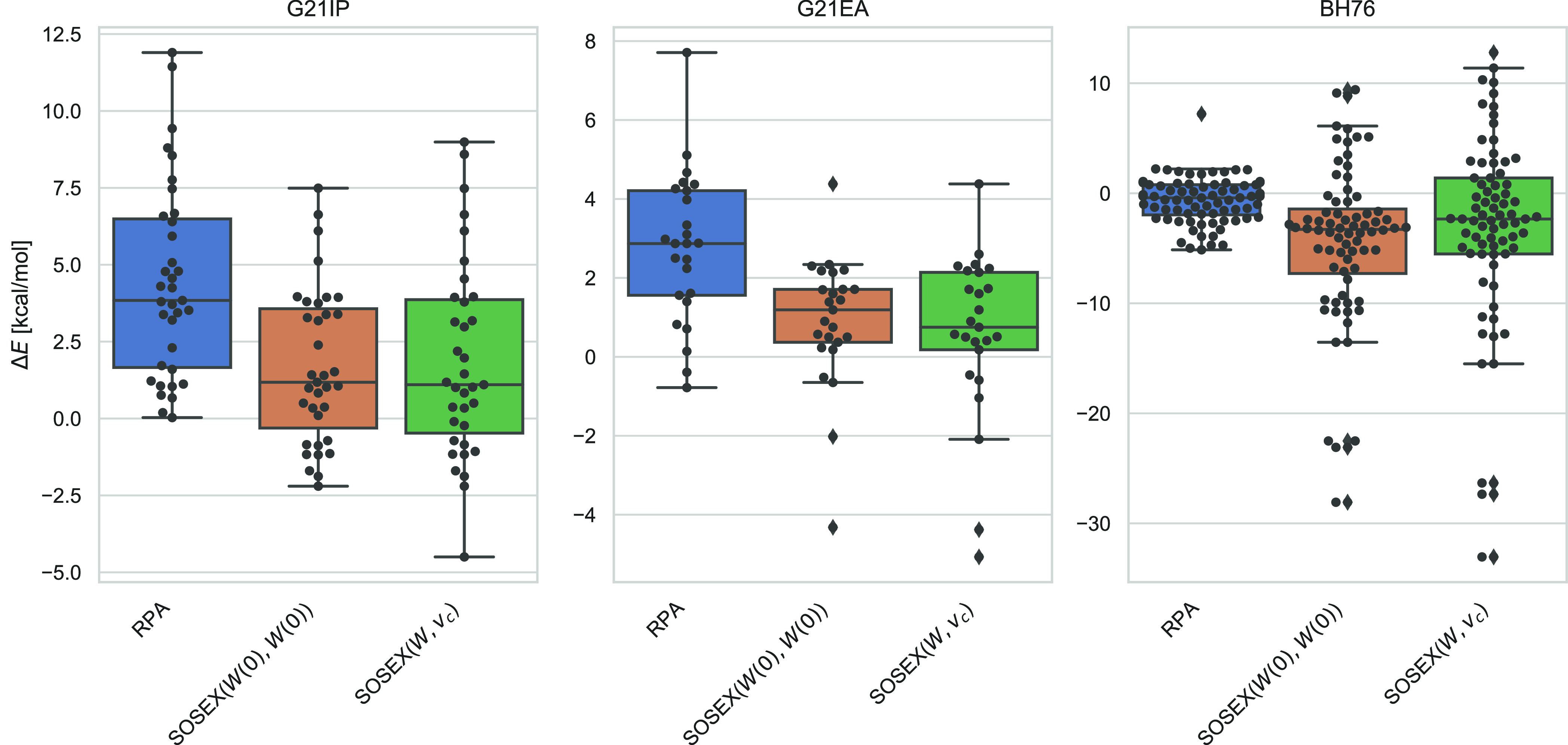
Errors of RPA@PBE and different RPA+SOSEX variants for barrier heights (BH76, left), ionization potentials (G21-IP, middle), and electron affinities (G21-EA, right). For an explanation of the boxplots, see the caption of Figure 4. All values are in kcal/mol.

As also shown in more detail in Figure 5, the performance of RPA+SOSEX(*W*(0), *W*(0)) is in all cases comparable to RPA+SOSEX(*W*, *v*_*c*_), for which the trends presented here are well-known:^5,6,87,112,172^ RPA+SOSEX(*W*, *v*_*c*_), fails for barrier heights, where the inclusion of renormalized singles excitations is necessary to obtain good results,^6,86,87^ and works very well for charged excitations.^5,6^ We note, that RPA+SOSEX(*W*(0), *W*(0))@PBE0 performs very well for charged excitations, with an accuracy challenging modern double-hybrid functionals.^149^

### Noncovalent Interactions

#### S66 Interaction Energies

**Figure 6:**
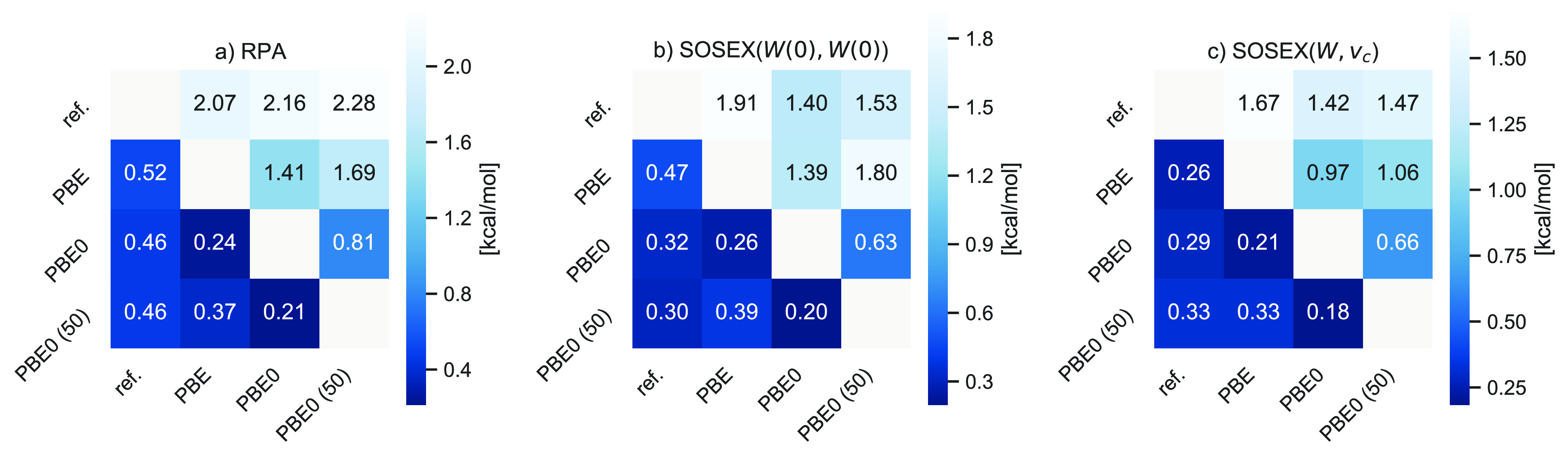
Mean absolute deviations (MAD) (lower triangle in each plot) and maximum deviations (MAX) (upper triangle) for (a) RPA, (b) SOSEX(*W*(0), *W*(0)), and (c) SOSEX(*W*(0), *v*_*c*_) interaction energies for the S66 database using different KS Green’s functions as well as to the CCSD(T) reference values (ref). All values are in kcal/mol.

We now turn to our benchmark results for noncovalent interactions. As for the previous data sets, we also assess the dependence of RPA and RPA+SOSEX correlation energies on the choice of the KS Green’s function *G*^*s*^. In Figure 6 the interaction energies in the S66 database^173^ obtained using different *G*^*s*^ are compared to each other as well as to the CCSD(T) reference values by Hobza and co-workers.^173^ All values have been obtained using a single integration point for the λ-integral. As shown in the Supporting Information, a few outliers aside the errors arising from this approximation are small for noncovalent interactions. RPA and RPA+SOSEX(*W*(0), *W*(0)) are equivalently independent of the choice of the KS Green’s function, with MADs between 0.20 and 0.39 kcal/mol between the different functionals. However, individual values can differ by almost 2 kcal/mol which is a sizable difference, given that the largest interaction energies in the S66 database are of the order of 20 kcal/mol only. The performance of RPA compared to the CCSD(T) reference is rather insensitive to the KS Green’s function, even though the hybrid starting points lead to slightly better results.^174^ The RPA+SOSEX(*W*(0), *W*(0)) results are much better using the hybrid functionals than with PBE. RPA+SOSEX(*W*, *v*_*c*_)@PBE is slightly more accurate than RPA+SOSEX(*W*, *v*_*c*_)@PBE0, but unlike for the data sets discussed before, the differences between the different starting points are negligibly small. Also, the dependence of SOSEX(*W*, *v*_*c*_) on the starting point is smaller than for SOSEX(*W*(0), *W*(0)).

**Figure 7:**
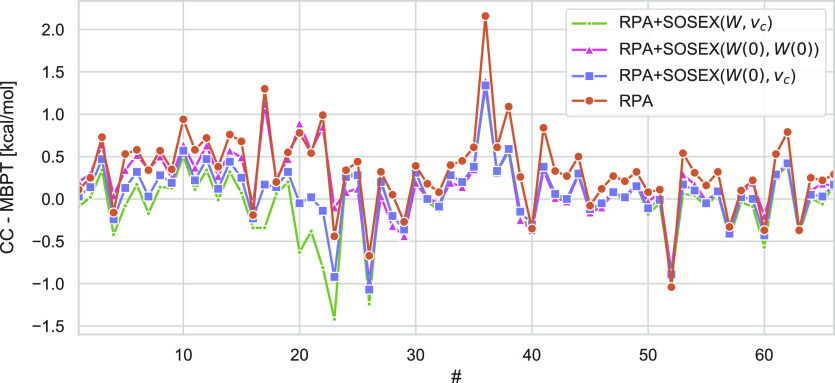
Deviations of RPA@PBE0 and both RPA + SOSEX@PBE0 variants for the S66 database with respect to the CCSD(T) reference. All values are in kcal/mol.

Figure 7 shows the deviations of RPA and both RPA+SOSEX variants with respect to CCSD(T) for all data points in the S66 database. MADs and mean absolute percentage deviations (MAPD) for the whole database as well as for the individual categories are presented in Table 3. The interactions of the first 22 complexes in the database are dominated by hydrogen bonds which are predominantly of electrostatic origin.^131^ The next 22 complexes are mostly bound by dispersion interactions and the remaining interactions are of mixed nature.^173^ It is useful to distinguish between these different interaction patterns in the following comparison.

**Table 3:** MADs (Absolute and in %) of Different Electronic Structure Methods with Respect to the CCSD(T) Reference Values for the Whole S66 Database and for Its Subcategories

	MAD							
	S66		hydr. bond		dispersion		mixed	
Method	[kcalmol]	[%]	[kcalmol]	[%]	[kcalmol]	[%]	[kcalmol]	[%]
SOSEX(*W*(0), *W*(0))@PBE0	0.32	7.28	0.45	5.76	0.29	10.33	0.21	5.50
SOSEX(*W*(0), *v*_*c*_)@PBE0	0.28	6.88	0.30	3.42	0.34	11.77	0.20	5.25
SOSEX(*W*, *v*_*c*_)@PBE0	0.29	6.85	0.31	3.39	0.33	11.63	0.21	5.33
SOSEX(*W*, *v*_*c*_)@PBE	0.26	6.25	0.23	3.51	0.33	10.16	0.17	4.26
RPA	0.46	11.54	0.55	7.19	0.47	17.74	0.34	9.41
PBE0-D3(BJ)	0.28	5.09	0.47	4.80	0.18	5.09	0.18	5.42
DSD-PBE-P86-D3(BJ)	0.23	5.07	0.31	3.71	0.21	6.99	0.16	4.43

For the whole database, RPA gives a MAPD of 11.5% and the SOSEX corrections sizably reduce the MAPDs with respect to the CCSD(T) reference values to in between 7.3% and 6.3%. SOSEX(*W*, *v*_*c*_) outperforms SOSEX(*W*(0), *W*(0)) by far for the hydrogen-bonded complexes, and is even slightly more accurate than the double-hybrid DSD-PBE-P86-D3(BJ),^175^ one of the best double hybrid functionals for weak interactions.^176^ For dispersion interactions, the performance of SOSEX(*W*(0), *W*(0)) and SOSEX(*W*, *v*_*c*_) is comparable. Here, the empirically dispersion-corrected^177,178^ functionals, the hybrid PBE0-D3(BJ) and DSD-PBE-P86-D3(BJ), are much more accurate than all MBPT based methods. A few exceptions aside, Figure 7 shows that RPA understabilizes the complexes in the S66 database (indicated by positive errors). SOSEX corrections lower the interaction energies, that is, the complexes are predicted to be more stable. SOSEX(*W*, *v*_*c*_) shows a tendency to overstabilize the hydrogen-bonded complexes. For these systems, the RPA+SOSEX(*W*(0), *W*(0)) energies are almost identical to the ones from RPA.

Also from the sizable differences of SOSEX(*W*, *v*_*c*_) (green points) to its static limit (with only a single screened interaction line, blue points) shown in Figure 7 it is clear that the dynamical screening effects are important for the hydrogen-bonded complexes. As can be seen from the MAPD in Table 3, this does however not improve agreement with the CCSD(T) reference values. For the dispersion bound complexes, there are only negligible differences between both variants, demonstrating that the dynamical variations of the screening average out. For the last 22 complexes in the database, the differences are slightly larger. In all cases, addressing the second electron–electron interaction line does not alter the results decisively.

#### S66 × 8 Interaction Energy

The S66 × 8 data set contains the complexes in the S66 database at 8 different geometries.^173^ The separations of the monomers in the complexes are given relative to their equilibrium distances; that is, a relative separation of 2.0 means that the monomers separation in the complex is twice as large as the equilibrium separation. For our assessment of the SOSEX(*W*(0), *W*(0)) correction, we divide the separations of the potential energy curve in three regions, which we denote as short (equilibrium distance scaled by a factor 0.9–0.95), middle (1.0–1.25), and long (1.5–2.0). All RPA (+SOSEX) calculations discussed here have been performed using a PBE0 Green’s function.

**Figure 8:**
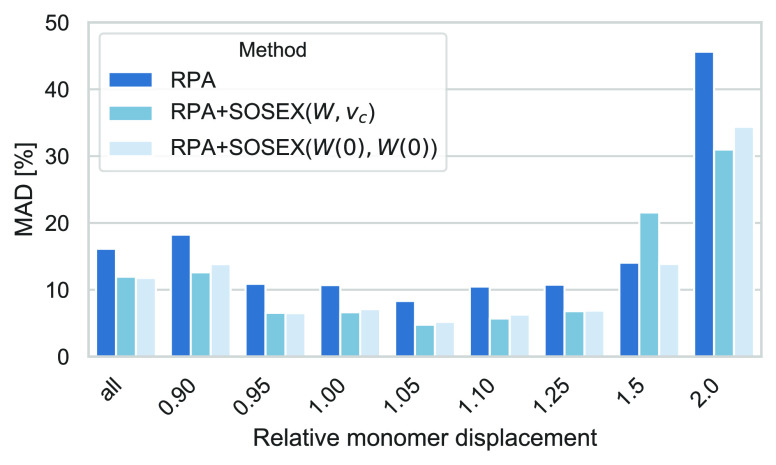
MADs (in percent) for the S66 × 8 database with respect to the CCSD(T) reference values for RPA, RPA+SOSEX(*W*, *v*_*c*_), and RPA+SOSEX(*W*(0), *W*(0)). MADs are shown separately for the whole database (columns on the left) and for different monomer–monomer separations.

**Figure 9:**
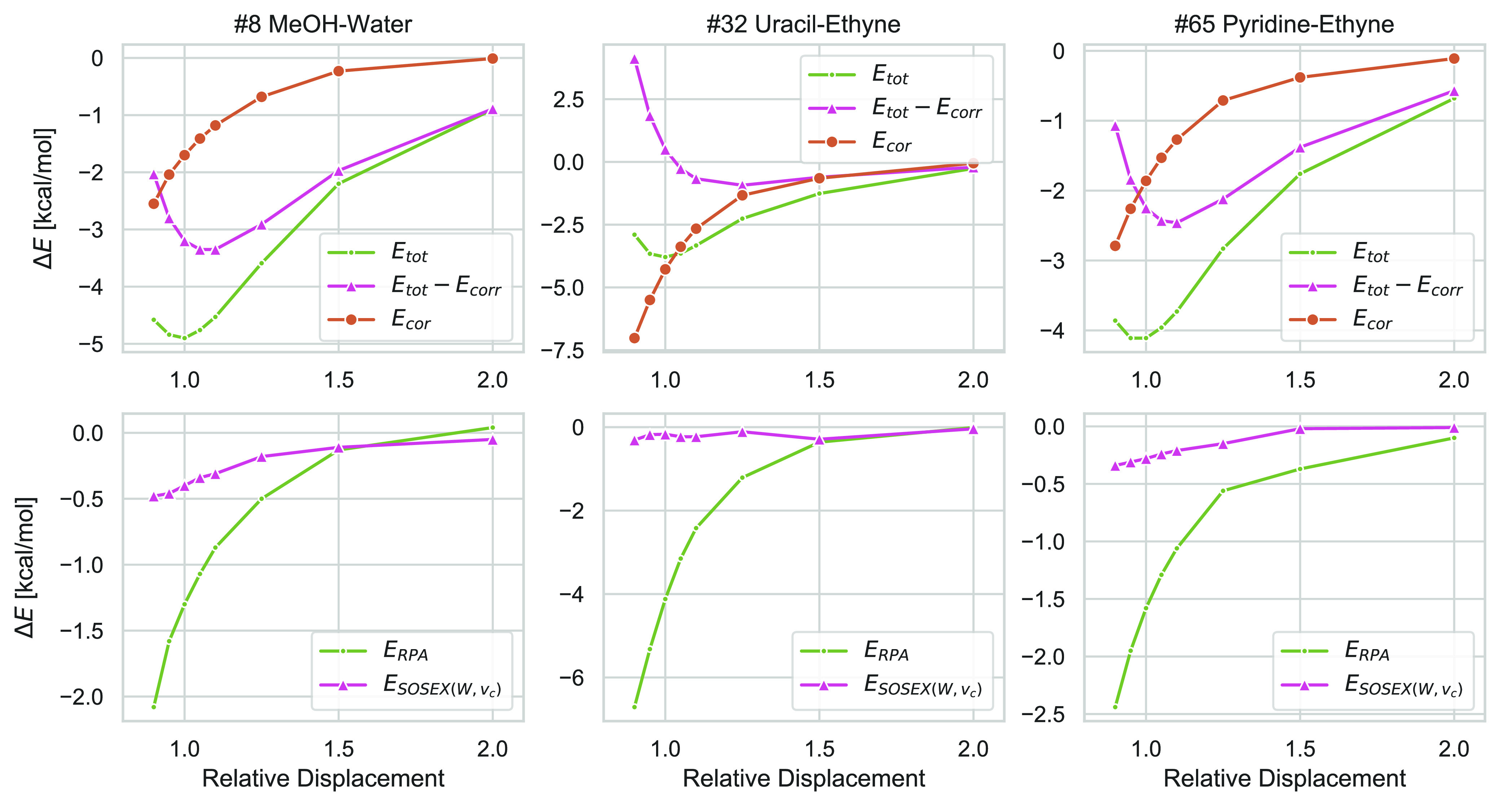
Upper plots: three RPA+SOSEX(*W*, *v*_*c*_)@PBE0 potential energy curves in the S66 × 8 database (green), separated in correlation contributions (yellow) and HF energy (evaluated with PBE0 orbitals). Lower plots: decomposition of the correlation energies into RPA and SOSEX(*W*, *v*_*c*_) contributions. All values are in kcal/mol.

The results of our comparison are shown in Figure 8, where the MAPDs with respect to CCSD(T) for the whole database as well as for the scaled monomer–monomer separations are shown. For the whole database, the average relative deviations with respect to the reference values are larger than for S66 (Table 4). With in between 31 and 43%, both SOSEX corrections lead to sizable improvements over the RPA in the short and medium regime. For large monomer–monomer separations, the improvements become much smaller, with 14% for SOSEX(*W*, *v*_*c*_) and 19% for SOSEX(*W*(0), *W*(0)). This can be rationalized by observing that for large electron–electron distances the correlation contributions to the interaction energies quickly decay to zero. This is shown in Figure 9 where we have plotted three of the RPA+SOSEX(*W*, *v*_*c*_) potential energy curves (green curves in the upper plots) in the S66 × 8 database and separated the correlation contributions. (The Green curves are the sums of the red and yellow curves.) The lower plots separately show the RPA and SOSEX(*W*, *v*_*c*_) contributions to the correlation energy differences.

**Table 4:** Relative Improvements Obtained with Different SOSEX Variants over RPA for Different Groups of Monomer–Monomer Separations

	short [%]	middle [%]	long [%]
SOSEX(*W*, *v*_*c*_)	35.2	42.8	13.5
SOSEX(*W*(0), *W*(0))	31.0	37.9	19.1

In all three plots, the potential energy curves are dominated by the difference of the correlation energy of the dimer and the sum of correlation energies of the monomers. Therefore, the approximation used for the calculation of the correlation energy plays a large role. However, this difference quickly goes to zero for larger separations. At two times of the equilibrium distance, the correlation contributions to the potential energy curves are almost zero in all three considered examples. Therefore, the expression used for the correlation energy becomes less and less important with increasing monomer separation. This argument also holds if one expresses the contributions in % of the total interaction energy.

One would expect the SOSEX contribution to decay faster than the RPA one, since the former is of exchange nature and therefore fundamentally short-ranged.^52^ However, the plots in the lower part of Figure 9 show that this is only the case for the potential energy curve on the right, but not for the two curves on the left, where SOSEX and RPA contributions seem to decay equally fast.

## 5 Conclusions

The accuracy of the RPA can in principle be improved by including vertex correction in the self-energy. This can be done either directly, or indirectly through the solution of the BSE. Different variants of SOSEX are obtained by including the first-order vertex in the self-energy. These are the well-known AC-SOSEX, herein termed SOSEX(*W*, *v*_*c*_), first introduced by Jansen et al.,^111^ in which only one of the Coulomb interaction lines is dynamically screened, as well as an energy expression which is obtained from the statically screened *G*3*W*2 correction to the *GW* self-energy.^114,115^ This energy expression has already been introduced in our earlier work,^114^ albeit without a rigorous derivation. Especially, we have implicitly assumed that the integral over the coupling strength is evaluated using a trapezoidal rule. Here, we have derived this expression (referred to as SOSEX(*W*(0), *W*(0)) in this work) taking into account its λ-dependence and highlighted the differences to SOSEX(*W*, *v*_*c*_). We have then assessed the accuracy of the SOSEX(*W*(0), *W*(0)) correction to RPA correlation energies for a wide range of chemical problems including bond dissociation, thermochemistry, kinetics, and noncovalent interactions.

The main conclusion we can draw from our work is that in a situation where the addition of SOSEX(*W*, *v*_*c*_) leads to major improvements over the RPA, the addition of SOSEX(*W*(0), *W*(0)) does so as well. This is the case for the calculation of ionization potentials and electron affinities where RPA+SOSEX approaches challenge the accuracy of modern double-hybrid functionals.^149^ Also for noncovalent interactions both SOSEX variants lead to the same substantial improvements over RPA. SOSEX(*W*, *v*_*c*_) is most accurate for the hydrogen-bonded complexes while SOSEX(*W*(0), *W*(0)) is slightly more accurate for dispersion interactions. We also showed that the frequency-dependence of the screened interactions does seem to be an important factor for hydrogen-bonding but not for dispersion interactions.

Differences between both SOSEX variants have been observed in the dissociation of diatomic molecules. As RPA and unlike RPA+SOSEX(*W*, *v*_*c*_),^112,145^ RPA+SOSEX(*W*(0), *W*(0)) dissociates the hydrogen molecule correctly. RPA does so because the self-correlation error effectively describes static correlation.^145^ The situation seems to be similar for RPA+SOSEX(*W*(0), *W*(0)) since in contrast to RPA+SOSEX(*W*, *v*_*c*_) it is not completely self-correlation free for 1-electron systems. We have also shown that this qualitative difference is due to the screening of the second electron–electron interaction line.

The incomplete cancellation of self-correlation error does however negatively affect the dissociation of charged dimers for which RPA+SOSEX(*W*, *v*_*c*_) fixes most of the deficiencies of RPA.^112,150^ Here, RPA+SOSEX(*W*(0), *W*(0)) performs even worse than RPA. Furthermore, the good dissociation of diatomic molecules does not automatically carry over to accurate barrier heights^153,154^ where both SOSEX variants considerably worsen the RPA results.

In summary, our results suggest that the statically screened SOSEX is a suitable alternative to dynamically screened SOSEX. While both formally scale as *N*^5^ with system size, the computation of the SOSEX(*W*, *v*_*c*_) correction requires a numerical imaginary frequency integration. The calculation of the SOSEX(*W*(0), *W*(0)) correction is therefore much less expensive, comparable to MP2. MP2 is however inadequate for large molecules since it neglects screening effects entirely.^1,48^ RPA+SOSEX(*W*(0), *W*(0)) is in principle applicable also to large molecules. A stochastic linear scaling implementation of the SOSEX self-energy has already been developed^179^ and a recent RPA+SOSEX implementation by Ochsenfeld and co-workers^180^ allowed applications to the L7 data set,^181^ albeit with small basis sets. Other low-scaling MP2 implementations^182−184^ could potentially be generalized to SOSEX as well.

Finally, it should be mentioned that the accuracy of the dynamically screened SOSEX correction to the RPA can be improved upon by the addition of renormalized single excitations.^6,87^ Other methods which have been shown to outperform SOSEX, in particular for barrier heights, are the AXK kernel method^94,150,185^ or a SOSEX variant in which the terms of RPA and SOSEX beyond second order in *v*_*c*_ are scaled down.^185^ It remains to be investigated whether the concept of static screening can also be combined with those approaches and leads to good results.

## Supporting Information Available

All calculated energies at the extrapolated CBS TZ3P, and QZ6P level (XLSX)
Detailed derivation and discussion of eq 6 for the self-energy, discussion of the crossing symmetries of the 4-point vertices, assessment of the dependence of the λ-integration on the number of Gauss-Legendre points for the S66 database (PDF)
