# Supplementary material for: Assessment of the Second-Order Statically Screened Exchange Correction to the Random Phase Approximation for Correlation Energies
Source: J Chem Theory Comput. 2022 Sep 23;18(10):5948–65. doi: 10.1021/acs.jctc.2c00366 (PMC9558381; doi:10.1021/acs.jctc.2c00366)
Supplement: Supplementary file 2 — ct2c00366_si_002.pdf [file ct2c00366_si_002.pdf]

Supporting information to: Assessment of the second-order statically screened exchange correction to the random phase approximation for correlation energies

Arno Förster*

Theoretical Chemistry, Vrije Universiteit, De Boelelaan 1083, NL-1081 HV, Amsterdam, The Netherlands

E-mail: a.t.l.foerster@vu.nl

A Crossing Symmetries of the 4-particle Vertices

One can show (see ref. 1 or ref. 2 for details) that the crossing symmetry of G_2 translates into the requirement

$$\frac{\delta\Sigma(1,3)}{\delta G(4,2)} \stackrel{!}{=} \frac{\delta\Sigma(2,4)}{\delta G(3,1)} \; , \tag{1}$$

for the 4-point vertex. In the GWA, the irreducible vertex is approximated with the Hartree-vertex,[3]

$$\Gamma^{(0)}_{Hxc}(1,2,3,4) \approx \Gamma^{(0)}_{H}(1,2,3,4) = -i\frac{1}{\delta G(1,3)}\delta(1,3)\int d2\, W^{(0)}(1,2)G(2,2^+) \; . \tag{2}$$

The functional derivative is

$$i\frac{\delta\Sigma_H(1,3)}{\delta G(4,2)} = \delta(1,3)\delta(2,4)W^{(0)}(1,2) \tag{3}$$

and therefore (1) is clearly violated when the 4-point vertex is approximated by the functional derivative of the Hartree contribution of the self-energy alone. However, when the 4-point vertex is approximated by the functional derivative of the Hartree-exchange self-energy the crossing symmetry is fulfilled: the functional derivative of the exchange-contribution to the vertex is

$$i\frac{\Sigma_x(1,3)}{\delta G(4,2)} = \delta(1,4)\delta(3,2)W^{(0)}(1,3) = -\delta(1,4)\delta(3,2)W^{(0)}(1,2)\ , \tag{4}$$

and therefore

$$\begin{aligned} i\frac{\Sigma_{Hx}(1,3)}{\delta G(4,2)} =& W^{(0)}(1,2)\left[\delta(1,3)\delta(2,4) - \delta(1,4)\delta(3,2)\right] \\ =& W^{(0)}(1,2)\left[\delta(1,4)\delta(2,3) - \delta(1,3)\delta(2,4)\right] = i\frac{\Sigma_{Hx}(2,4)}{\delta G(3,1)}\ . \end{aligned} \tag{5}$$

B The Self-Energy in terms of the 4-point Vertex

Since the expression for the self-energy used in the main document,

$$\Sigma_{xc}(1,2) = iG(1,2)W(1,2) + iG(1,3)W(1,4)\chi^{(0)}(6,4,5,4^+)\Gamma^{(0)}_{xc}(6,5,2,3)\ , \tag{6}$$

is not commonly used in quantum chemistry, we derive it in this appendix from the expression for the single-particle Green's function in terms of the 2-particle Green's function,

$$G(1,1') = G^{(0)}(1,1') - iG^{(0)}(1,2)W^{(0)}(2,3)G_2(2,3^+,1',3^{++})\ , \tag{7}$$

which follows directly from the Heisenberg equation-of-motion for the field operator.[4] Using

$$\chi(1,2,3,4) = -iG_2(1,2,3,4) - iG(1,2)G(3,4) \tag{8}$$

we can write this as

$$
\begin{aligned}
G(1,1') =& G^{(0)}(1,1') - iG^{(0)}(1,2)W^{(0)}(2,3)G(2,1')G(3,3^{+}) \\
& - G^{(0)}(1,2)W^{(0)}(2,3)\chi(2,3^{+},1',3^{++}) \; .
\end{aligned}
\tag{9}
$$

the second term on the *r.h.s* is the first term in the series expansion of G in terms of the bare interaction. The corresponding contribution to the self-energy is the Hartree term,

$$
\Sigma_H(1,2) = \delta(1,2)v_H(1) = -i\delta(1,2)\int d3W^{(0)}(1,3)G(3,3+) \; . \tag{10}
$$

Eq. (9) becomes

$$
\begin{aligned}
G(1,1') =& G^{(0)}(1,1') + G^{(0)}(1,2)\Sigma_H(2,3)G(3,1') \\
& - G^{(0)}(1,2)W^{(0)}(2,3)\chi(2,3^{+},1',3^{++}) \; .
\end{aligned}
\tag{11}
$$

We now consider the single-particle Green's function in the presence of the time-dependent source term,

$$
\begin{aligned}
G_u(1,1') =& G^{(0)}(1,1') + G^{(0)}(1,2)\left[u(2,3) + \Sigma_H(2,3)\right]G_u(2,1') \\
& + iG^{(0)}(1,2)W^{(0)}(2,3)\frac{\delta G_u(2,1')}{\delta u(3^{+},3)} \; .
\end{aligned}
\tag{12}
$$

Following ref. 4, this is equivalent to

$$
\begin{aligned}
G_u^{-1}(1,1') =& \left[G^{(0)}\right]^{-1}(1,1') - \left[u(1,1') + \Sigma_H(1,1')\right] \\
& - iW^{(0)}(2,3)\frac{\delta G_u(1,2)}{\delta u(3^{+},3)}G_u^{-1}(2,1') \; ,
\end{aligned}
\tag{13}
$$

and by comparison to the Dyson equation of the unperturbed interacting system, the self-energy is

$$
\Sigma(1,1') = \Sigma_H(1,1') - iW^{(0)}(1,3)G(1,2)\frac{\delta G^{-1}(2,1')}{\delta u(3^{+},3)} \; , \tag{14}
$$

where the last factor in the last term becomes (using (12) in the first equation and (8) as well as the expression for the 4-point vertex,

$$\Gamma^{(0)}_{Hxc}(1,2,3,4) = \Gamma^{(0)}_{H}(1,2,3,4) + \Gamma^{(0)}_{xc}(1,2,3,4) = i\frac{\delta\Sigma_H(1,3)}{\delta G(4,2)} + i\frac{\delta\Sigma_{xc}(1,3)}{\delta G(4,2)} \ , \tag{15}$$

in the last equation)

$$\begin{aligned}
\frac{G^{-1}(2,1')}{\delta u(3^+,3)} =& \frac{\left[G^{(0)}\right]^{-1}(2,1')}{\delta u(3^+,3)} - \frac{u(2,1')}{\delta u(3^+,3)} - \frac{\Sigma(2,1')}{\delta u(3^+,3)} \\
=& -\delta(2,1')\delta(2,3) - \frac{\delta\Sigma(2,1')}{\delta u(3^+,3)} \\
=& -\delta(2,1')\delta(2,3) - \frac{\delta\Sigma(2,1')}{\delta G(4,4')}\frac{\delta G(4,4')}{\delta u(3^+,3)} \\
=& -\delta(2,1')\delta(2,3) - \Gamma^{(0)}(2,4',1',4)\chi(4,3,4',3^+) \ .
\end{aligned} \tag{16}$$

Inserting the last equation into (14) we identify the Fock term

$$\Sigma_x(1,1') \equiv iW^{(0)}(1,3)G(1,2)\delta(2,1')\delta(2,3) = iW^{(0)}(1,1')G(1,1') \ . \tag{17}$$

The remainder is called correlation term,

$$\Sigma_c(1,1') \equiv +iW^{(0)}(1,3)G(1,2)\Gamma^{(0)}(2,4',1',4)\chi(4,3,4',3^+) \ , \tag{18}$$

$$\Sigma(1,1') = \Sigma_H(1,1') + \Sigma_x(1,1') + \Sigma_c(1,1') \ . \tag{19}$$

We now split the 4-point vertex as

$$\begin{aligned}
\Gamma^{(0)}(1,2,1',2') &= \Gamma^{(0)}_H(1,2,1',2') + \Gamma^{(0)}_{xc}(1,2,1',2') \\
&= i\frac{\delta\Sigma_H(1,1')}{\delta G(2',2)} + i\frac{\delta\Sigma_{xc}(1,1')}{\delta G(2',2)} \ .
\end{aligned} \tag{20}$$

Using eq. (2) and ?? in (18) gives

$$\begin{aligned}\Sigma_c(1,1') =& iG(1,2)W^{(0)}(1,3)\chi(4,3,4,3^+)W^{(0)}(4,1') \\ &+ iG(1,2)W^{(0)}(1,3)\chi(4,3,4',3^+)\Gamma^{(0)}_{xc}(2,4',1',4)\end{aligned} \tag{21}$$

and collecting all terms, we obtain for the total self-energy

$$\begin{aligned}\Sigma(1,1') =& \Sigma_H(1,1') + iW^0(1,1')G(1,1') \\ &+ iG(1,1')W^{(0)}(1,2)\chi(4,3,4,3^+)W^{(0)}(4,1') \\ &+ iG(1,1')W^{(0)}(1,3)\chi(4',3,4,3^+)\Gamma^{(0)}_{xc}(2,4,1',4') \;.\end{aligned} \tag{22}$$

This expression is equivalent to the expression given by Maggio and Kresse in ref. 5 and also to eq. 10.37 in ref. 4. Since

$$\chi(4,3,4,3^+) = -iG(4,3^+)G(3,4) = P(4,3) = P(3,4) \tag{23}$$

and using the definition of the screened electron-electron interaction in terms of the interacting polarizability P

$$W(1,2) = W^{(0)}(1,2) + W^{(0)}(1,3)P(3,4)W^{(0)}(3,2) \tag{24}$$

we can combine the second and third term on the *r.h.s.* of (22). This is the GW contribution to the self-energy. We then finally obtain (6)

$$\Sigma(1,2) = \Sigma_H(1,2) + iG(1,2)W(1,2) + iG(1,3)W^{(0)}(1,4)\chi(6,4,5,4^+)\Gamma^{(0)}_{xc}(3,5,2,6) \;. \tag{25}$$

Notice, that we have renamed the variables in the last term for better readability. The equivalence of this expression to Hedin's expression for the self-energy[6] has for instance been shown in references 7 and 5. In case screening is calculated within the RPA, χ is related

to its non-interacting counterpart $\chi^{(0)}$ by a Dyson equation with the bare electron-electron interaction as its kernel,

$$\chi(1,2,3,4) = \chi^{(0)}(1,2,3,4)\chi^{(0)}(1,5,3,6)W^{(0)}(5,7,6,8)\delta(5,6)\delta(7,8)\chi(8,2,7,4) \; . \quad (26)$$

Then

$$\begin{aligned} W^{(0)}\chi =& W^{(0)}\left[\chi^{(0)} + \chi^{(0)}W^{(0)}\chi^{(0)} + \chi^{(0)}W^{(0)}\chi^{(0)}W^{(0)}\chi^{(0)} + \dots\right] \\ =& \left[W^{(0)} + W^{(0)}\chi^{(0)}W^{(0)} + W^{(0)}\chi^{(0)}W^{(0)}\chi^{(0)}W^{(0)} + \dots\right]\chi^{(0)} \\ =& W\chi^{(0)} \; . \end{aligned} \quad (27)$$

eq. (25) can then also be written as

$$\Sigma(1,2) = \Sigma_H(1,2) + iG(1,2)W(1,2) + iG(1,3)W(1,4)\chi^{(0)}(6,4,5,4^+)\Gamma^{(0)}_{xc}(3,5,2,6) \; , \quad (28)$$

where the unscreened electron-electron interaction $W^{(0)}$ does not appear any more. This is the expression from which we have derived the expressions for the different variants of the SOSEX self-energy in the main text.

C λ-dependence of the S66 Interaction Energies

We herein discuss the dependence of the SOSEX correlation energies on the λ-integration. The magnitude of the SOSEX contribution to the correlation energy as a function of the size of the λ-grid is shown in table 1 for three selected systems. One can show, that for a 2-electron system like H_2, the SOSEX(W, v_c) correction equals minus half of the RPA correlation energy (In other words, RPA+SOSEX is self-correlation free). This relation is fulfilled for SOSEX(W, v_c) with already 4 Gauss-Legendre points.

The magnitude of the SOSEX correction is underestimated when the λ-integration is carried out using a trapezoidal rule. This is illustrated in fig. 1 for H_2 and $(H_2O)_2$. Using a single λ-point corresponds to approximating the λ-dependence of the correlation energy as a

Table 1: Total magnitude of SOSEX correction in kcal/mol as a function of the size of the λ-grid for selected systems.

	SOSEX(W, v_c)			SOSEX$(W(0), W(0))$		
N_λ	H_2	$(H_2O)_2$	Benzene	H_2	$(H_2O)_2$	Benzene
1	15.51400	147.15253	287.90735	10.74228	101.45448	204.17377
2	16.63810	157.79684	307.48549	12.83037	119.99186	239.22546
4	16.63421	157.71007	307.35326	12.82006	119.79484	238.91728
6	16.63421	157.70997	307.35313	12.82006	119.79451	238.91688
$\frac{1}{2}E^{RPA}$	16.63421			16.63421		

straight line, which leads to a small integration error. In the supporting information we show the effect of the number of points in the λ-integration on the relative energies in the S66 dataset to be indeed very small. Also the accuracy of the interaction energies with respect to the CCSD(T) reference values is not negatively affected when only a single integration point is used and therefore all benchmark results for the S66x8 database are carried out using the trapezoidal rule.

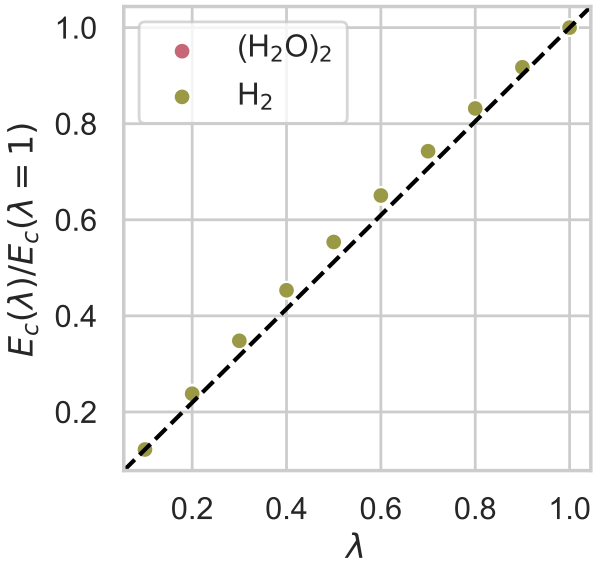

Figure 1: Magnitude of the SOSEX(W, v_c) correlation energy as a function of λ relative to its value at $\lambda = 1$ for H_2 and $(H_2O)_2$.

The effect of the number of points in the λ-integration on relative energies for SOSEX(W, v_c) and SOSEX$(W, W(0))$ for the S66 test set is shown in figure 2. Fig. 2a) shows the error in the

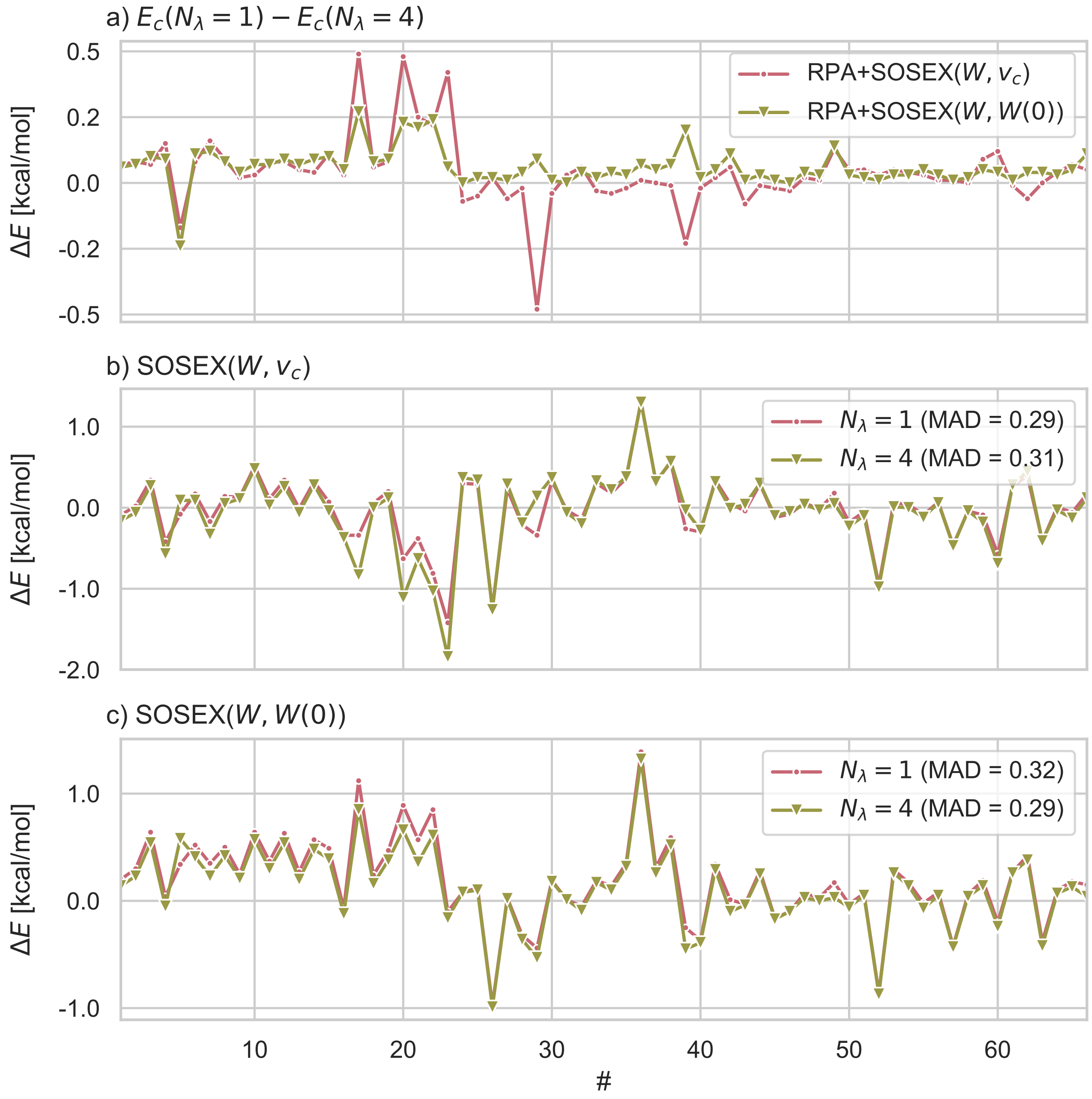

Figure 2: a) Difference in relative correlation energies for the S66 test set for both SOSEX variants when using 1 and 4 integration points for the λ-integration. b) Error of relative SOSEX(W, v_c) correlation energies with respect to the CCSD(T) reference values when using 1 and 4 integration points for the λ-integration. c) Same as b), but for SOSEX($W, W(0)$). All values are in kcal/mol.

covalent bonding energies with respect to the converged $\lambda-$integration when only a single integration point is used. Generally, the resulting integration errors are very small and do not exceed 0.1 kcal/mol for most complexes. In parts b) and c) of figure 2, the relative errors

of the interaction energies with respect to the CCSD(T) reference values are shown. One can clearly see, that the error in the λ-integration is negligible when looking at the accuracy of relative energies. This is also reflected in the MADs with respect to the CCSD(T) reference shown in figure 2.

D Energy differences

All calculated energies are collected in .xlsx files. These files are organized as follows:

This file contains all counterpoise corrected interaction energies calculated for this work. In each file:

- the second column contains the reference interaction energy for each complex,
- columns 3-5 contain the interaction energies calculated at the TZ3P, QZ6P, and extrapolated CBS level.
- Columns 6-8 contain the deviation to the reference energies:
- Column 6 contains the deviation of column 2 and 3, and so on

References

(1) Rohringer, G.; Valli, A.; Toschi, A. Local electronic correlation at the two-particle level. *Phys. Rev. B - Condens. Matter Mater. Phys.* **2012**, *86*, 125114.

(2) Rohringer, G.; Hafermann, H.; Toschi, A.; Katanin, A. A.; Antipov, A. E.; Katsnelson, M. I.; Lichtenstein, A. I.; Rubtsov, A. N.; Held, K. Diagrammatic routes to nonlocal correlations beyond dynamical mean field theory. *Rev. Mod. Phys.* **2018**, *90*, 25003.

(3) Romaniello, P.; Guyot, S.; Reining, L. The self-energy beyond GW: Local and nonlocal vertex corrections. *J. Chem. Phys.* **2009**, *131*, 154111.

(4) Martin, R. M.; Reining, L.; Ceperley, D. M. *Interacting electrons*; Cambridge University Press, 2016.

(5) Maggio, E.; Kresse, G. GW Vertex Corrected Calculations for Molecular Systems. *J. Chem. Theory Comput.* **2017**, *13*, 4765–4778.

(6) Hedin, L. New method for calculating the one-particle Green's function with application to the electron-gas problem. *Phys. Rev.* **1965**, *139*, A796.

(7) Starke, R.; Kresse, G. Self-consistent Green function equations and the hierarchy of approximations for the four-point propagator. *Phys. Rev. B* **2012**, *85*, 075119.
